# Assessment of Alcohol and Tobacco Use Disorders Among Religious Users of Ayahuasca

**DOI:** 10.3389/fpsyt.2018.00136

**Published:** 2018-04-24

**Authors:** Paulo Cesar Ribeiro Barbosa, Luís F. Tófoli, Michael P. Bogenschutz, Robert Hoy, Lais F. Berro, Eduardo A. V. Marinho, Kelsy N. Areco, Michael J. Winkelman

**Affiliations:** ^1^Department of Philosophy and Human Sciences, Universidade Estadual de Santa Cruz, Ilhéus, Brazil; ^2^Department of Medical Psychology and Psychiatry, Faculty of Medical Sciences, Universidade Estadual de Campinas, Campinas, Brazil; ^3^New York School of Medicine, New York, NY, United States; ^4^University of New Mexico, Simpson Hall, Albuquerque, NM, United States; ^5^Department of Psychiatry and Human Behavior, University of Mississippi Medical Center, Jackson, MS, United States; ^6^Department of Health Sciences, Universidade Estadual de Santa Cruz, Ilhéus, Brazil; ^7^Departamento de Psiquiatria, Universidade Federal de São Paulo, São Paulo, Brazil; ^8^School of Human Evolution and Social Change, Arizona State University, Tempe, AZ, United States

**Keywords:** *ayahuasca*, hoasca, União do Vegetal, alcohol, tobacco, substance use disorder

## Abstract

The aims of this study were to assess the impact of ceremonial use of *ayahuasca*—a psychedelic brew containing N,N-dimethyltryptamine (DMT) and β-carboline —and attendance at *União do Vegetal* (UDV) meetings on substance abuse; here we report the findings related to alcohol and tobacco use disorder. A total of 1,947 members of UDV 18+ years old were evaluated in terms of years of membership and ceremonial attendance during the previous 12 months. Participants were recruited from 10 states from all major regions of Brazil. Alcohol and tobacco use was evaluated through questionnaires first developed by the World Health Organization and the Substance Abuse and Mental Health Services Administration. Analyses compared levels of alcohol and tobacco use disorder between the UDV and a national normative sample (*n* = 7,939). Binomial tests for proportions indicated that lifetime use of alcohol and tobacco was higher in UDV sample compared to the Brazilian norms for age ranges of 25–34 and over 34 years old, but not for the age range of 18–24 years old. However, current use disorders for alcohol and tobacco were significantly lower in the UDV sample than the Brazilian norms. Regression analyses revealed a significant impact of attendance at *ayahuasca* ceremonies during the previous 12 months and years of UDV membership on the reduction of alcohol and tobacco use disorder.

## Introduction

Alcohol and tobacco substance-related disorders and health consequences are a major public health problem. The World Health Organization [1] estimated that the global prevalence of alcohol use disorder (AUD) in 2010 was 4.1%, including alcohol dependence (2.3%) and harmful use of alcohol (1.8%). The WHO also estimated that 3.3 million (or 5.9%) of 2012 global deaths were attributable to alcohol use. Alcohol causes or negatively impacts several health conditions, including neuropsychiatric impairment, gastrointestinal diseases, cancers, cardiovascular diseases, and fetal and birth complications [1]. Tobacco related health conditions such as cancer, cardiovascular diseases, and respiratory system diseases account for 12% of deaths of people aged 30 and over around the globe [2].

Despite the substantial amount of resources that official organizations invest in programs to prevent substance use and its adverse consequences, substance-related problems are still a major public health issue across the globe [1, 3, 4]. Current treatment options for alcohol and tobacco disorders include an impressive diversity of pharmacological, psychosocial, and technological resources that are nonetheless only partially effective [5–8]. Consequently further investigations are needed to explore and evaluate new strategies to prevent and treat substance-related disorders.

During the last two decades evidence has emerged that suggests that *ayahuasca*, a psychedelic brew containing N,N-dimethyltryptamine (DMT) and β-carboline, has effective properties to reduce substance abuse. Originally used for magico-religious purposes by Amerindian populations of the Amazon Basin, modern syncretic forms of ritual *ayahuasca* use have spread to major Brazilian cities and to many other countries. Currently *ayahuasca* is used within formal religions, such as União do Vegetal (UDV), Santo Daime, and Barquinha; in a worldwide distribution of “*vegetalistas*,” particularly from Peru; as well as in religious-independent contexts, such as *ayahuasca* retreats.

The first rigorous evidence that *ayahuasca* could have positive effects on substance-related problems was published by Grob et al. [9], who reported the absence of current drug/alcohol-related problems in a sample of UDV members (where *ayahuasca* is referred to as *hoasca*). They used the Composite International Diagnostic Interview (CIDI) to assess 15 adult long-term UDV members and 15 non-*ayahuasca*-user controls. Interestingly, this research found evidence for past diagnostic criteria for alcohol use disorders (AUD) for 5 UDV members who no longer met the criteria after becoming UDV members. In comparison, only one control subject showed past and no longer active AUD diagnosis. Accordingly, Doering-Silveira et al. [10] found lower past month and past year alcohol use in 41 adolescents who were ayahuasca users from the UDV compared to 43 matched control adolescents with no previous *ayahuasca* exposure. Another research group administered the Addiction Severity Index 5th Edition (ASI-5) and found lower recent use of alcohol among 127 adult *ayahuasca* users from the Santo Daime and Barquinha relative to 75 non-*ayahuasca* users [11]. A recent study administered the ASI-5 to compare substance use among 30 UDV *ayahuasca* users with 27 non-*ayahuasca* users who were active members of Catholic and Protestant churches [12]. Relative to the control group, the UDV group demonstrated greater past use of alcohol to intoxication and past use of cannabis, but less recent use of alcohol. In a cross-sectional evaluation of 32 Santo Daime members with no control group, Halpern et al. [13] administered the Structured Clinical Interview for DSM-IV psychiatric disorders and detected an individual with marijuana dependence in partial remission and another subject with ongoing marijuana abuse. However, 22 participants with a previous history of drug/alcohol-related problems were in full remission. Thomas et al. [14] conducted a prospective study of 12 participants with substance-use related problems who attended an *ayahuasca* retreat and subsequently reported decreased cocaine, tobacco and alcohol use. Finally, Lawn et al. [15] conducted a cross-sectional survey with 527 participants who had used *ayahuasca* in the past year. Their participants ingested *ayahuasca* in settings as diverse as a “Healing centre,” “Retreat,” “Ceremony,” “Church,” “Santo Daime,” “Teacher,”and “Trained Facilitator.” Out of the 527 participants, 192 were using *ayahuasca* for the first time. The authors compared *ayahuasca* users with users of other psychedelics (a group who had used psychedelic mushrooms and LSD, but not *ayahuasca*, in the last year, *n* = 18,138) and with non-psychedelic users (a group who had no exposure to *ayahuasca*, LSD or psychedelic mushrooms in the last year, *n* = 78,236). Using the Alcohol Use Disorder Identification Test (AUDIT) as a measure, the authors reported less problematic use of alcohol in the *ayahuasca*-using group during the past year than reported by the other psychedelic group, but more problematic use of alcohol in the *ayahuasca* group than the non-psychedelic using respondents.

In order to contribute to the literature on the potential therapeutic effects of religious *ayahuasca* use on substance use disorder, the present study investigated substance use patterns among a much larger sample of *ayahuasca* users than those of the aforementioned studies. We recruited volunteers who were members of the UDV from 10 Southern, Southeastern, Central, Northeastern, and Northern Brazilian States. The prevalence of alcohol and tobacco use and disorder in the UDV sample was analyzed in comparison with the prevalence in a Brazilian normative sample. We also evaluated the association of alcohol and tobacco use and disorders with years of UDV membership and *ayahuasca* ceremonial attendance during the previous 12 months.

### Background information: União do Vegetal

The *União do Vegetal* (UDV) has been as described as a “Brazilian *ayahuasca* religion”, which also includes other groups such as Santo Daime, Barquinha and other groups that use *ayahuasca* in their ceremonies [16, 17]. The religious use of *ayahuasca* is legal in Brazil and loosely regulated by the Brazilian government [17–19]. The UDV is known for its greater organizational capacity and mobilization relative to the other *ayahuasca* religions. It has a book edited by a sociologist member [20] and a Scientific Commission that analyzes, and in some cases (such as in the present study), authorizes and provides logistic support to studies done in the institution. In 2012, there were estimated to be approximately 16,500 UDV members in South and North America and Europe. In addition to the ritual use of *ayahuasca*, these religions subscribe to Christian principles, as well as beliefs regarding reincarnation. The UDV is especially restrictive regarding the use of alcohol, tobacco, and illicit substances.

The *ayahuasca* consumed by the members of the group is under formal ritualized conditions. Special ceremonies are held to prepare the *ayahuasca*, where the two officially recognized ingredients—the bark of the *Banisteriopsis caapi* vine and the leaves of *Psychotria* species are boiled together in water. Regular *ayahuasca* sessions (*escala*) open to all members and invited visitors are held twice a month, but higher ranking members (*mestres, conselheiros*, and *corpo instructivo*) may drink the brew more frequently in additional closed meetings (see [21] for additional details).

The *ayahuasca*-induced modification of consciousness is thought to stimulate spiritual growth, moral development and the evolution of consciousness, embodied in their motto “Light, Peace and Love.” Regular ceremonies start at 8:00 p.m. with the attendees approaching the leader (*mestre*) in an order of rank, waiting in line to individually receive a dose in a glass (150–250 ml). The amount of ceremonial doses received by each participant is determined by the *mestre*, in consultations with senior members, particularly those who personally know the person; these consultations are generally via short utterances or non-verbal signs. Attendees may also be queried regarding their preferred dose or if they want more. The preaching occurs through *mestre* sermons, questions directed to the preacher by the participants to the *mestre*, through popular songs with moral contents played on stereo equipment and also through hymns performed by single participants. During some periods silence predominates (see [21] for additional details). An optional additional dose of the brew is available until 10 PM. A short break occurs approximately 3 ½ h into the ceremony, with a brief return to conclude the approximately 4 h ceremony by midnight. The ceremony is followed by consumption of a light meal and socializing that may last for hours.

## Methods

### Design

A cross-sectional study surveyed 1,947 UDV volunteers between March 2009 and August 2011. Participants were recruited from 35 UDV temples of 10 Brazilian states from the Country's five major administrative areas: Amazon and Pará states (North); Bahia and Ceará states (Northeast); Mato Grosso, Mato Grosso do Sul, Goiás and Distrito Federal states (Central); São Paulo state (Southeast); and Santa Catarina (South). Efforts were made to exhaustively survey the members attending the church during the activities on a single day at each local.

### Recruitment procedures

Firstly, we obtained a preliminary authorization from UDV central leadership and approval from UDV Scientific Committee to conduct the survey in its local temples. The UDV's Scientific Committee (*Comissão Cient*í*fica da UDV*) and Medical Scientific Department (*Departamento Medico Cient*í*fico*—DEMEC) then contacted local UDV temples to explain the study goals and gather a list of volunteers. Volunteers could choose to fill out hard copy or online versions of the questionnaires. We code numbered the volunteers‘list so that personal names were separated from questionnaires in order to assure confidentiality and anonymity. Church attendees received envelopes and questionnaires according to their ID number. The members were informed about the survey by the local officers of the DEMEC, who explained to them that participation in the study was voluntary, i.e., only those who wanted to participate in the survey should fill out the questionnaires. Those who opted for the online version received an ID number to insert in the corresponding field of the questionnaire website. Both the electronic and hard paper versions of the questionnaire contained a cover letter explaining the aims of the study and the consent form that explained the voluntary nature of their participation; it also explained the confidentiality and anonymity of the volunteer's information, and the planned assessment on substance use disorder. The volunteers who agreed to participate signed two copies of the consent form and mailed one to the researchers in a postage-paid envelope provided. The study was approved by the Universidade Federal de Santa Catarina Committees on the Ethics of Human Research (Process #244/08 FR - 216544).

### Instruments

#### Socio-economic variables

Age, sex, years of education, and marital and employment status were solicited.

#### Church membership and patterns of ayahuasca use

This questionnaire evaluated several dimensions concerning the frequency of ritual attendance during the previous 12 months and the total years of UDV membership.

#### SAMHSA-assessed substance use disorder and WHO criteria

The data for our comparison group for alcohol and tobacco use disorder was based on the II Household Survey on the use of psychotropic drugs in Brazil: 2005 [22]. This survey was conducted by the Centro Brasileiro de Informações sobre Drogas Psicotrópicas (CEBRID) of the Universidade Federal de São Paulo (UNIFESP) and the Secretaria Nacional Antidrogas (SENAD), the Brazilian federal agency responsible for coordinating actions of the national drug policy. This household survey assessed a sample of 7,939 subjects throughout Brazil to estimate the Brazilian prevalence of substance use disorders (SUD) in four age ranges: 12–17, 18–25, 26–34, and over 34 years old. The SUD was assessed by using a Brazilian version of the Substance Abuse and Mental Health Services (SAMHSA) [23, 24] questionnaire [22]. The questionnaire has six questions regarding: (1) the length of time spent to get the drug or for recovering from its effects; (2) substance use in larger quantities or more often than intended; (3) tolerance (i.e., need to use larger doses of the drug in order to experience the same effect); (4) physical risks (e.g., driving and swimming under the effect of the drug); (5) personal problems (e.g., family and work problems); and (6) the desire to diminish or cease the use of the drug. Positive answers to two or more these questions would indicate the presence of the substance use disorder.

We also administered the alcohol and tobacco sections of a questionnaire based on the WHO Research and Reporting Project on the Epidemiology of Drug Dependence [25]. The questionnaire has been adapted for Brazilians [26, 27] and extensively used for the evaluation of patterns of drug use among Brazilian students [28]. The instrument is a self-report questionnaire containing multiple-choice questions about lifetime drug use and during the last 12 months and last month. Participants who answer positively to lifetime use of a specific drug (i.e., if they were exposed to the substance at least once in their lifetime) were asked about its at least once use during the last 12 months; those who answer positively to the use in the last 12 months are then asked about its use during the past month. In addition, we added a question regarding substance use during the year before joining UDV.

In addition to the use of alcohol and tobacco reported here, SAMHSA and WHO questionnaires also assessed the use of other substances, including marijuana, hallucinogens, and cocaine. Our report on the use of these other substances among UDV members is underway.

#### Statistical analyses

Statistical analyses were performed using IBM SPSS 20.0 for Windows. Binomial tests for proportions were used to assess the difference between the UDV sample and Brazilian norms for lifetime use of substances and SAMSHA criteria of substance use disorder. We tested the difference between the Brazilian normative sample and the whole UDV sample, as well as comparison of the Brazilian norms with a subsample of UDV members who had been active members of the religion for more than 3 years. Hierarchical logistic regression was used to assess the prediction of the *ayahuasca* ceremony attendance variables on dichotomous dependent variables regarding SAMSHA criteria for substance disorder and use of substances during the previous 30 days and previous 12 months. The first model included age, gender and level of education as explanatory variables. The second model added *ayahuasca* ritual attendance variables, which were the frequency of *ayahuasca* ceremonies during the previous 12 months and a binary variable regarding years of church membership, distinguishing members with up to 3 years of UDV membership from members with more than 3 years of UDV membership. The decision to transform the continuous variable years of UDV membership into a binary variable resulted from preliminary graphical analyses, which demonstrated that the cut-off point of 3 years of membership would have more explanatory power in the logistic models. Multi-colinearity of independent variables was assessed via Pearson' and Spearman's rho correlation matrixes and Variance Inflation Factor (VIF). We set potential co-linearity problems values of 0.7 for Pearson' and Spearman's correlations and over 10 for VIF. Level of significance for variables contributing to the model was set at *p* < 0.05.

## Results

### Sociodemographic profile

The respondents were predominantly from the central, south and southeast regions of Brazil (68.7%). They had a mean age of 39.85 years (range 18–81) and were evenly distributed between males (50.8%) and females (49.2%). The majority of the respondents (68.4%) were married or in a stable relationship. They had a mean of 9.44 years of UDV membership and a mean of 34.99 ceremonies attended within the last year (see Tables 1, 2 for details).

**Table 1:** Sociodemographic variables.

**AGE**			
Mean (St. Dev)			39.85 (SD = 12.078)
Median			38.00
Min- Max			18–81
*N*			1,902
		***N***	**Valid%**
**NUMBER OF SUBJECTS**			
Men		982	50.8
Women		952	49.2
	Total	1,934	100.0
**DEGREE OF EDUCATION**			
< Bachelor's		907	47.2
> Bachelor's		1,013	52.8
	Total	1920	100.0
**MARITAL STATUS**			
Single/Never been married		447	23.1
Stable cohabiting with partner		278	14.4
Married		1,043	54.0
Separated/Divorced		134	6.9
Widow		29	1.5
	Total	1,931	100.0
**EMPLOYMENT STATUS**			
Student		215	11.3
Employed		458	24.1
Public Servant		404	21.3
Self-employed		479	25.2
Stay-at-Home		139	7.3
Employer		126	6.6
Unemployed		14	0.7
Retired		66	3.5
	Total	1,901	100.0
**BRAZILIAN GEOGRAPHIC AREAS**			
Central area: Mato Grosso do Sul, Mato Grosso, Goiás, Distrito Federal		674	34.6
South and Southeast areas: São Paulo, Santa Catarina		663	34.1
Northeast area: Ceará, Bahia		342	17.6
North area: Pará, Amazonas		267	13.7
	Total	1,946	100.0

**Table 2:** Ayahuasca ritual attendance variables.

	***N* (valid %)**	**Mean**	**St. Dev**.	**Median**	**Min**	**Max**
Years of UDV membership	1,765	9.4486	7.716	7.25	0.01	41.42
≤3 years	372 (21.1%)					
> 3 years	1,393 (78.9%)					
Frequency of ayahuasca ritual attendance during the previous 12months	1,772	34.99	12.714	34.00	3	120
Days abstinent from ayahuasca	1,868	6.92	8.253	5.00	0	120

### Comparison between UDV sample and brazilian norms for lifetime use of alcohol and tobacco

Binomial tests for proportions indicated that lifetime use of alcohol and tobacco was higher in the UDV sample compared to the Brazilian norms for age ranges of 25–34 and over 34 years old, but not for the age range of 18–24 years old. All differences were statistically significant (*p* < 0.001), except for the comparison between lifetime tobacco use for the age range of 18–24 years old (Table 3).

**Table 3:** Lifetime use of alcohol and tobacco.

**Age range**	**UDV**	**Brasil**	***p***	***O.R*.**
	***% (a)***	***% (b)***		
**ALCOHOL**				
18–24	72.2	78.6	0.029	0.71
25–34	86.6	79.5	<0.001	1.66
≥35	87.7	75.0	<0.001	2.39
**TOBACCO**				
18–24	34.9	39.5	0.126	0.82
25–34	52.8	40.8	<0.001	1.62
≥35	61.3	52.6	<0.001	1.43

### Comparison between the UDV sample and the brazilian norms for current alcohol and tobacco use disorder

UDV sample showed significantly lower prevalence of alcohol and tobacco use disorder compared to the Brazilian norms for all age ranges. When Brazilian norms were compared with the UDV subsample with more than 3 years of UDV membership, those differences were of a larger magnitude (Table 4).

**Table 4:** Current alcohol and tobacco use disorder.

**Age range**	**UDV**	**Brasil**	***p***	***O.R*.**
	***% (a)***	***% (b)***		
**ALCOHOL**				
18–24	4.9	19.2	<0.001	0.22
25–34	2.3	14.7	<0.001	0.14
≥35	1.0	10.4	<0.001	0.08
**ALCOHOL—UDV SUBSAMPLE 3 YEARS MEMBERSHIP**				
18–24	3.4	19.2	<0.001	0.15
25–34	0,5	14.7	<0.001	0.03
≥35	0.2	10.4	<0.001	0.02
**TOBACCO**				
18–24	2.4	9.4	<0.001	0.24
25–34	2.5	9.4	<0.001	0.24
≥35	0.9	12.2	<0.001	0.06
**TOBACCO—UDV SUBSAMPLE 3 YEARS MEMBERSHIP**				
18–24	1.6	9.4	<0.001	0.16
25–34	1.1	9.4	<0.001	0.10
≥35	0.4	12.2	<0.001	0.03

### Hierarchical logistic regressions for alcohol use

Table 5 shows the results for the hierarchical logistic regression analyzes for alcohol use variables. There was no evidence of multicollinearity for any of the logistic regression models, as assessed by VIF greater than 10, nor evidence of multicollinearity as assessed by Pearson's and Spearman's correlations greater than 0.7. The covariates age, gender and level of education included in the first model (Model 1) explained very little of the variance of alcohol use during the previous 12 months (2.2%; *p* = 0.001) and previous 30 days (2.8%; *p* = 0.009), and a little more of the variance of alcohol use disorder (7%; *p* = 0.001). Gender did not significantly contribute to the first model (model 1) for any of the alcohol variables. Age was negatively associated (OR: 0.974, 95% CI 0.959 to 0.989; *p* = 0.001) with use of alcohol during the previous 12 months, whereas having a Bachelor's degree was positively associated (OR: 1.555, 95% CI 1.090 to 2.219; *p* = 0.015) with the use of alcohol during this period. Having a Bachelor's degree was also positively associated with the use of alcohol during the previous 30 days (OR: 2.599, 95% CI 1.394 to 4.846; *p* = 0.003), and age was negatively associated with alcohol use disorder (OR: 0.918, 95% CI 0.874 to 0.965; *p* = 0.001).

**Table 5:** Hierarchical logistic regressions for alcohol use.

**ALCOHOL USE DURING THE PREVIOUS 12 MONTHS**						
	**Model 1**			**Model 2**		
	**(*****N*** = **1,575; No** = **1,426; Yes** = **149)**			**(*****N*** = **1,562; No** = **1,420; Yes** = **142)**		
	**OR (95% CI)**		**Sig**.	**OR (95% CI)**		**Sig**.
**Variables**						
Age	0.974	(0.959–0.989)	0.001	0.991	(0.975–1.007)	0.266
Gender	0.871	(0.619–1.226)	0.430	0.755	(0.523–1.090)	0.133
Degree of Education > Bachelor's	1.555	(1.090–2.219)	0.015	1.791	(1.222–2.626)	0.003
Ceremonial attendance during the previous 12 months				0.936	(0.917–0.954)	< 0.001
Membership > 3 years				0.226	(0.154–0.332)	< 0.001
**Model summary**						
Na R^2^		0.022			0.205	
Δ R^2^		–			0.183	
**ALCOHOL USE DURING THE PREVIOUS 30 DAYS**						
	**Model 1**			**Model 2**		
	**(*****N*** = **1,572; No** = **1,517; Yes** = **55)**			**(*****N*** = **1,570; No** = **1,516; Yes** = **54)**		
	**OR (95% CI)**		**Sig**.	**OR (95% CI)**		**Sig**.
**Variables**						
Age	0.982	(0.958–1.006)	0.148	0.993	(0.968–1.018)	0.566
Gender	0.817	(0.474–1.408)	0.468	0.648	(0.366–1.150)	0.139
Degree of Education > Bachelor's	2.599	(1.394–4.846)	0.003	2.819	(1.479–5.374)	0.002
Ceremonial attendance during the previous 12 months				0.907	(0.880–0.936)	< 0.001
Membership > 3 years				0.401	(0.221–0.727)	0.003
**Model summary**						
Na R^2^		0.028			0.183	
Δ R^2^		–			0.155	
**ALCOHOL USE DISORDER**						
	**Model 1**			**Model 2**		
	**(*****N*** = **1,559; No** = **1,536; Yes** = **23)**			**(*****N*** = **1,559; No** = **1,536; Yes** = **23)**		
	**OR (95% CI)**		**Sig**.	**OR (95% CI)**		**Sig**.
**Variables**						
Age	0.918	(0.874–0.965)	0.001	0.946	(0.900–0.993)	0.026
Gender	0.949	(0.413–2.180)	0.902	0.854	(0.364–2.007)	0.718
Degree of Education > Bachelor's	0.977	(0.417–2.291)	0.957	1.071	(0.446–2.571)	0.878
Ceremonial attendance during the previous 12 months				0.946	(0.904–0.991)	0.018
Membership > 3 years				0.169	(0.062–0.464)	0.001
**Model summary**						
Na R^2^		0.070			0.184	
Δ R^2^		–			0.114	

*Na, Nagelkerke*.

The inclusion of *ayahuasca* ritual attendance variables in the second model (Model 2, see Table 5) increased the explanation of variance in alcohol use in the previous 12 months (NagR^2^ = 20.5%, Δ = 18.3%; *p* < 0.001), the variance explained alcohol use during the previous 30 days (NagR^2^ = 18.3%, Δ = 15.5%; *p* < 0.001), and the variance explained in alcohol use disorder (NagR^2^ = 18.4%, Δ = 11.4%; *p* < 0.001). The more the members attended UDV sessions during the previous 12 months, the less likely they were to have used alcohol during the previous 12 months (OR: 0.936, 95% CI 0.917 to 0.954; *p* < 0.001), during the previous 30 days (OR: 0.907, 95% CI 0.880 to 0.936; *p* < 0.001) or meet the criteria for alcohol use disorder (OR: 0.946, 95% CI 0.904 to 0.991; *p* = 0.018). Participants with more than 3 years of UDV membership were less likely to use alcohol during the previous 12 months (OR: 0.226, 95% 0.154 to 0.332; *p* < 0.001), during the previous 30 days (OR: 0.401, 95% 0.221 to 0.727; *p* = 0.003) or to meet criteria for alcohol use disorder (OR: 0.169, 95% 0.062 to 0.464; *p* = 0.001) than the participants with 3 years or less of UDV membership. In Models 2, having a Bachelor's degree increased the likelihood of using alcohol during the previous 12 months (OR: 1.760, 95% 1.202 to 2.578; *p* = 0.003) and during the previous 30 days (OR: 2.819, 95% 1.479 to 5.374; *p* = 0.002) in comparison to those members without bachelor's degree. Age was also negatively associated with criteria for alcohol use disorder (OR: 0.946, 95% 0.900 to 0.993; *p* = 0.026).

### Hierarchical logistic regressions for tobacco use

The covariates age, gender and level of education explained 1% (*p* = 0.292) of tobacco use in the previous 12 months, 1.2% (*p* = 0.488) of its use during the previous 30 days, and 0.6% (*p* = 0.745) of tobacco use disorder. None of these three variables significantly contributed to any of Model 1 analyses (Table 6). The Model 2 analyses with inclusion of ritual attendance measures (see Table 6) increased the variance explained in tobacco use during the previous 12 months (NagR^2^ = 11.6%, Δ = 10.6%; *p* < 0.001), the variance explained in the use of tobacco during the previous 30 days (NagR^2^ = 8.6%, Δ = 7.4%; *p* = 0.004) and the variance explained in tobacco use disorder (NagR^2^ = 9.1%, Δ = 8.5%; *p* = 0.002). Attendance at UDV ceremonies during the previous 12 months was negatively correlated with tobacco use in the same period (OR: 0.944, 95% CI 0.914 to 0.975; *p* < 0.001), negatively correlated with tobacco use during the previous 30 days (OR: 0.951, 95% CI 0.908 to 0.996; *p* = 0.033) and negatively correlated with tobacco use disorder (OR: 0.950, 95% CI 0.908 to 0.995; *p* = 0.029). Members with more than 3 years of UDV membership were less likely to have used tobacco during the previous 12 months (OR: 0.279, 95% 0.145 to 0.536; *p* < 0.001), less likely to have used tobacco during the previous 30 days (OR: 0.308, 95% CI 0.118 to 0.809; *p* = 0.017) and less likely to meet criteria for tobacco use disorder (OR: 0.258, 95% 0.100 to 0.667; *p* = 0.005) than the members with three years or less of membership in the UDV.

**Table 6:** Hierarchical logistic regressions for tobacco use during the previous 12 months, previous 30 days and tobacco use disorder.

**TOBACCO USE DURING THE PREVIOUS 12 MONTHS**						
	**Model 1**			**Model 2**		
	**(*****N*** = **1,581; No** = **1,536; Yes** = **45)**			**(*****N*** = **1,581; No** = **1,536; Yes** = **45)**		
	**OR (95% CI)**		**Sig**.	**OR (95% CI)**		**Sig**.
**Variables**						
Age	0.990	(0.965–1.016)	0.459	1.006	(0.980–1.032)	0.666
Gender	0.656	(0.355–1.213)	0.179	0.593	(0.315–1.113)	0.104
Degree of Education > Bachelor's	0.749	(0.410–1.369)	0.348	0.790	(0.426–1.465)	0.454
Ceremonial attendance during the previous 12 months				0.944	(0.914–0.975)	< 0.001
Membership > 3 years				0.279	(0.145–0.536)	< 0.001
**Model summary**						
Na R^2^		0.010			0.116	
Δ R^2^		–			0.106	
**TOBACCO USE DURING THE 30 DAYS**						
	**Model 1**			**Model 2**		
	**(*****N*** = **1,576; No** = **1,556; Yes** = **20)**			**(*****N*** = **1,576; No** = **1,556; Yes** = **20)**		
	**OR (95% CI)**		**Sig**.	**OR (95% CI)**		**Sig**.
**Variables**						
Age	1.020	(0.984–1.056)	0.285	1.032	(0.996–1.068)	0.080
Gender	0.745	(0.301–1.842)	0.524	0.703	(0.279–1.768)	0.453
Degree of Education > Bachelor's	0.645	(0.264–1.579)	0.337	0.676	(0.273–1.678)	0.399
Ceremonial attendance during the previous 12 months				0.951	(0.908–0.996)	0.033
Membership > 3 years				0.308	(0.118–0.809)	0.017
**Model summary**						
Na R^2^		0.012			0.086	
Δ R^2^		–			0.074	
**TOBACCO USE DISORDER**						
	**Model 1**			**Model 2**		
	**(*****N*** = **1,573; No** = **1,552; Yes** = **21)**			**(*****N*** = **1,573; No** = **1,552; Yes** = **21)**		
	**OR (95% CI)**		**Sig**.	**OR (95% CI)**		**Sig**.
**Variables**						
Age	0.987	(0.950–1.025)	0.489	1.004	(0.967–1.041)	0.853
Gender	1.207	(0.507–2.871)	0.671	1.132	(0.468–2.738)	0.783
Degree of Education > Bachelor's	0.741	(0.309–1.778)	0.502	0.777	(0.319–1.892)	0.578
Ceremonial attendance during the previous 12 months				0.950	(0.908–0.995)	0.029
Membership > 3 years				0.258	(0.100–0.667)	0.005
**Model summary**						
Na R^2^		0.006			0.091	
Δ R^2^		–			0.085	

*Na, Nagelkerke*.

## Discussion

In this study, the largest survey done with *ayahuasca* users to date, we found that UDV sample had remarkably lower rates of alcohol and tobacco use disorder relative to Brazilian norms, and that this difference was even greater when Brazilian normative sample was compared with the *ayahuasca* subsample with more than 3 years UDV membership. We also found that *ayahuasca* use variables—ceremonial attendance during the previous 12 months and years of UDV membership—were much stronger predictors of reduced alcohol and tobacco use disorders and use during the previous 12 months than were the SES variables age, gender and level of education.

Interestingly, the members of the UDV sample with more than 24 years of age had much greater lifetime exposure to tobacco and alcohol than the Brazilian normative sample. This finding is consistent with previous assessments of *ayahuasca* users, who have shown less current drug-related problems compared to controls, but more lifetime exposure to drugs [9, 11, 12]. This result, combined with the regression findings of the effect on ritual *ayahuasca* ceremonial attendance variables on lowering alcohol and tobacco use disorder, strongly support the hypothesis that ritual use of *ayahuasca* can have powerful therapeutic effects in addressing drug dependence problems.

The finding of Lawn et al. [15] that *ayahuasca* users had greater AUDIT-assessed problematic drinking than non-psychedelic users is a major exception to the emerging pattern of the negative association between *ayahuasca* intake and substance disorder [9–13]. This discrepancy may be due to the social support system provided by church membership, and suggests that the regularity of the use of *ayahuasca* within structured settings like UDV and Santo Daime play a important role in therapeutic and protective effects observed in the previous studies. In contrast, Lawn's et al. study included occasional *ayahuasca* users who took it in much more independent contexts (shamans, healers, retreats) than structured religious contexts, which may have influenced their less positive results. Furthermore, the UDV has a very restrict attitude toward alcohol and tobacco use, which may involve temporary interdiction of more advanced members who abuse these substances.

Religious variables are widely known for their strong protective and therapeutic effects on drug use and drug-related problems [29, 30]. The design of the current research does not allow for analyses the separate the effects of religious attendance from the pharmacological effects of *ayahuasca* on our positive findings. However previous studies suggest that the pharmacological effects of *ayahuasca*, rather than just the social dimensions of church support for sobriety, contributed substantially to these significant findings. This strictly pharmacological effect is suggested by a case-control study that found that UDV members had lower recent use of alcohol than a control group formed by active Christian church members [12]. Further support for strictly pharmacological mechanisms comes from a experimental study using animal models which found that *ayahuasca* inhibits ethanol-induced locomotion and prevents ethanol sensitization in mice [31]. Others suggest that the pharmacological mechanisms of the anti-addictive effects of *ayahuasca* involve mesolimbic dopaminergic pathways that are thought to underlie human craving and compulsive use of abused substances [32].

### Possible pharmacological mechanisms of ayahuasca's protective effects against substance abuse disorders

A variety of studies on *ayahuasca*-based substance abuse rehabilitation programs provide evidence of treatment efficacy (e.g., see [33–36]; also see [37] for review), although these studies fall short of the ideal double-blind clinical designs [38]. The constituents of *ayahuasca* provide a variety of physiological mechanisms by which it may reduce drug dependency (see [37, 38] for review). The therapeutic potential of *ayahuasca* in reducing addictive behaviors is likely mediated by the neurochemical aspects of both of its active components, DMT and β-carboline, that exert psychoactive properties in both humans and animals [39–41]. The classic pharmacokinetic model of *ayahuasca* involves the β-carboline alkaloids' inhibition of the biodegradation of DMT by monoamine oxidase A (MAO-A) in the gastrointestinal tract [42, 43]. By inhibiting monoamine oxidase, the β-carbolines allow DMT to reach systemic circulation in the central nervous system and exert its psychoactive effects. Furthermore, recent studies have shown that even β-carboline alkaloids alone can exert some therapeutic effects that lead to decreases in drug abuse-related behaviors in animal models (for review, see [41]). Thus, *ayahuasca's* complex and unique pharmacological profile provides multiple mechanisms through which it may exercise direct therapeutic effects for the treatment of drug dependence.

Liester and Prickett ([44]; also see [45]) propose that the treatment of the biochemical dynamics of addiction requires two effects, the first one on serotonin and the second one on dopamine. An increase in overall serotonin levels is first necessary to increase dopamine to levels sufficient to attenuate withdrawal symptoms. Subsequently there needs a normalization of dopamine levels that can be produced by inhibitory effects of serotonin on the mesolimbic dopamine pathways' release of dopamine. Liester and Prickett ([44, 45]) propose that *ayahuasca* produces both of these effects through a variety mechanisms, providing a neurochemical normalization therapy through pathways that both raise and modulate dopamine in the mesolimbic dopamine pathway (MDP), remedying addiction by first releasing sufficient dopamine to normalize dopamine levels, but then exerting an inhibitory influence that precludes an abrupt spiking in dopamine that can contribute to liability to addiction.

Because both DMT and β-carbolines are known to modulate brain serotonergic neurotransmission [41, 46, 47], the effects of *ayahuasca* on drug abuse may reflect the actions of its components at serotonin receptors. More specifically, DMT and β-carbolines appear to exert agonistic activity at serotonin 5-HT2A and 5-HT2C receptors [41, 46, 47]. Due to their distribution in the brain, 5-HT2A receptor activation increases dopamine levels in the nucleus accumbens (NAcc) [48], the main neurochemical effect by which drugs with abuse potential exert reinforcing and rewarding properties in animals and humans [49].

Based on this DMT-mediated effect on dopamine levels, *ayahuasca* would be expected to have abuse liability in humans. However, a literature review on *ayahuasca* has indicated that consumption of traditional preparations in social settings carries a minimal risk of abuse potential or dependence formation [50]. The lack of abuse liability, as well as the growing evidence of a therapeutic utility of ayahuasca for the treatment of drug abuse, further indicates that other mechanisms also play an important role in the psychoactive effects of ayahuasca. Of note, in contrast to what is observed with 5-HT2A receptor activation, 5-HT2C receptor agonists decrease NAcc dopamine levels [48], thereby antagonizing the dopamine-related effects of 5-HT2A receptor activation. In fact, 5-HT2C receptor agonists have been recently proposed as possible therapeutic targets for the treatment of drug addiction, including ethanol abuse [51–53].

This second anti-dependence mechanism of *ayahuasca* constituents is hypothesized to derive from the effects of the beta-carboline alkaloids that stimulate the release of dopamine in presynaptic neurons, consequently causing the release of both dopamine and serotonin in the MDP [44, 45]. While harmine can stimulate the release of dopamine, it also blocks dopamine reuptake into neurons at the synaptic membranes. Furthermore, DMT agonism action at the sigma-1 receptor receptors inhibits dopamine release, resulting in decreases in the dopamine spiking effects that reinforce addiction. These combined effects of *ayahuasca* constituents on MDP dopamine levels achieves a balance between the dopamine deficiencies that produce withdrawal symptoms and the spikes in dopamine levels that contribute to the formation of dependency.

Thus, because DMT and β-carbolines have been proposed to activate both receptor subtypes, a unique pattern of activation of 5-HT2A/2C receptors seems to underlie the therapeutic effects of *ayahuasca* and its lack of abuse liability. This dual action underlies the potential of *ayahuasca* as a therapy for drug-dependence through these combined effects on the MDP that are considered to be the common pathway underlying most if not all addictive drugs. The action of the beta-carbolines may also lead to increases serotonin levels through inhibition of MAO enzymes, blocking the enzymatic metabolism of catecholamines, and leading to augmentation of dopamine levels [45]. It is important to note, however, that both DMT and β-carbolines also interact with other non-serotonergic molecular targets, and a possible contribution of those to the effects of *ayahuasca* on drug abuse cannot be ruled out [41, 54].

The concept of neuroplasticity, the ability of neurons to alter their synaptic connections, provides additional physiologic mechanisms for effects against dependence exercised by *ayahuasca* [45]. Neuroplasticity is first involved in the addiction learning process, a maladaptive learning process that unfolds in the acquisition of habits of drug dependence, the patterns of learned behavior and association that contribute to the compulsion and reward cycles involved in the habitual self-administration of drugs.

Prickett and Liester [45] outline a variety of mechanisms are involved in the production of neuroplasticity. These include the elimination of synapses, formation of new synapses, a remodeling of dendrites and axons). *Ayahuasca* can affect neuroplasticity through a variety of neurochemical mechanisms that facilitate changes in neural architecture, including the disruption of learned associations that underlie addictive behaviors through responses to triggers and cues that were hardwired into the neural networks in the process of addiction. This *ayahuasca* effect may facilitate neurophysiologic changes that address the behavioral and neurochemical dynamics of addiction by causing a neurological rewiring in the brain's reward pathways.

### Set and setting: context effects in addressing dependence

However the mere use of *ayahuasca* alone may not be effective in reducing addictive behaviors; the importance of the set and setting in psychedelic effects is a well-established principal of the global mechanisms of action of these substances, implicating social factors as significant variables in the reduction of dependence. For example, the Takiwasi program [33] attributes the treatments effects of *ayahuasca* as derived not just from the physiological properties, but also from the ritual conditions and the social environment, especially the interaction of the patients' personal psychology with the therapists and other participants [33]. The program attributes a significant therapeutic role to the content of the visionary experiences that provides crucial information for the client, as well as the psychological dispositions involving openness to the process and a psychological surrender that manifests a commitment to the ritual treatment processes. Fernández and Fábregas propose that therapeutic efficacy of *ayahuasca* derives from, among other factors, the visionary experiences of one's past that provide insights into the origins of the addictive patterns of behavior, combined with other powerful emotional experiences, especially death experiences and transpersonal experiences that lead to a greater awareness of potential for personal transformation (also see [34]).

The UDV members have commented on similar dynamics as operating in the processes of remission of drug dependence, especially as expressed in the concept of “recognition of one's errors.” This alludes to the dynamic effects of *ayahuasca* in enhancing an awareness of one's personal psychological dynamics, connecting the patient with causal factors in their personal past that contributed to dependency, and elevating these repressed memories to consciousness. Acknowledgement of these errors of the past and the path to better relations with others reflect the pharmacological actions of *ayahuasca* constituents. The *ayahuasca* visions and recollections of the past are consequences of biological effects that contribute important therapeutic dynamics through connecting the patient with significant aspects of their personal past, elevating repressed memories into consciousness where they can play a role in psychological healing through restructuring. These consciousness-enhancing effects of *ayahuasca* may be the result of an increased activation of brain areas that enhance somatic awareness, emotional arousal, emotional processing (the right hemisphere areas of the anterior insula and anterior cingulate/frontomedial cortex and left hemisphere amygdala/parahippocampal gyrus structures) [55]. These brain area activations elevate repressed memories into consciousness, allowing for a novel reprocessing of these memories, particularly those associated with a traumatic past relations with family members. Human imaging studies [54] led McKenna and Riba [56] to postulated a model of *ayahuasca* effects involving increased neuronal excitability in brain areas involved in sensory, memory and emotional processing and inhibition of top-down constraints or expectations. Those effects modulate *ayahuasca*-induced visions, especially intense emotions and recollection of personal memories; this allows for the safe exposure to emotional events, similarly to cognitive and exposure therapies [54]. Evidence for such mechanisms of action involving *ayahuasca* are suggested by studies of cognitive training interventions that have shown promising results in reducing drug-related effects that are mediated by memory and emotional neuromechanisms, such as motivational salience of drug-associated stimuli, including the context of alcohol and tobacco abuse (for review see [57]).

Broadly assessed, these studies suggest that religious and psychological mechanisms, as well as pharmacological and neurophysiological mechanisms, are involved in the reductions in substance dependence among UDV members. Studies such as the present one suggest that we can expect promising results in reducing the effects of addictive drugs with the use of *ayahuasca* is supportive settings. The regular group interaction of the UDV members certainly constitutes a significant feature of the social support for sobriety and social condemnation for lapses. The twice monthly open religious meetings are supplemented for members with several other opportunities for consumption in settings for the advanced members in weekend retreat-like settings, providing further involvement in life when drug-use risks are higher.

Our finding that the history of consuming drugs is significantly higher in UDV members than in Brazilian population norms is noteworthy, as is their current status with considerably lower levels of consumption. These findings are recognized by the UDV members, who emphasize the importance of their group as a drug-treatment strategy, frequently citing the success of group membership in instilling abstinence in certain members. It appears that members' enthusiasm regarding the success of the group as a treatment for substance dependence may also extend to a recruitment tool for members of their social network who suffer such dependencies. Such outreach may account for the relatively high rates of alcohol and tobacco use reported by the members for the period prior to their church adherence. Another possible contributory factor to the high rates of prior dependence reported by UDV members is their desire to emphasize the effectiveness of their religious activity by overstating prior dependencies.

## Conclusions

Although the molecular and cellular interactions of *ayahuasca* are not yet fully established, a range of studies suggest that *ayahuasca* has a broad range of psychoactive effects that can modulate dependence in ways that reduce drug use and abuse patterns. The present study provides further evidence of those effects by showing that in a large population sample, current levels of alcohol and tobacco dependence were lower in *ayahuasca* users compared to the general population, even though previous drug use was higher among this group prior to becoming church members. Moreover, there was a substantial negative relation of the variables assessing ceremonial *ayahuasca* ingestion with the variables assessing substance use and disorder, demonstrating that the attendance and consumption of *ayahuasca* were likely the causal factors.

The cross-sectional design of the present study limits our ability to establish a causal relationship between the *ayahuasca* ceremonial attendance variables and lowering of substance use and substance disorders in the UDV sample. Moreover, the present design is vulnerable to self-selection and recall biases. The major strength of the present study is the sample size of *ayahuasca* users evaluated. Relative to the large scale survey concluded recently [15], our study has the advantage of having evaluated regular *ayahuasca* users, and of having analyzed the association between different degrees of ceremonial *ayahuasca* exposure with alcohol and tobacco use and disorder. Further research to establish the physiological mechanisms involved in reduction of substance abuse by *ayahuasca* should consider separate use of extracted DMT and harmaline compounds in double-blind assessments.

## Author contributions

Each of the authors participated in this research by contributing to the conception and design of the study PR, MW study management, PR, MW, and LT collecting data, PR, MW, and LT statistical analysis and interpretation PR, MW, LT, MB, RH, LB, EM, and KA and the preparation of the manuscript PR, MW, and LB.

### Conflict of interest statement

The authors declare that the research was conducted in the absence of any commercial or financial relationships that could be construed as a potential conflict of interest.
